# The Regenerative Role of the Fetal and Adult Stem Cell Secretome

**DOI:** 10.3390/jcm2040302

**Published:** 2013-12-17

**Authors:** Sveva Bollini, Chiara Gentili, Roberta Tasso, Ranieri Cancedda

**Affiliations:** Regenerative Medicine Laboratory, Department of Experimental Medicine (DIMES), University of Genoa, IRCCS AOU San Martino—IST, National Institute for Cancer Research, Largo Rosanna Benzi 10, Genoa 16132, Italy; E-Mails: sveva.bollini@hsanmartino.it (S.B.); chiara.gentili@unige.it (C.G.); roberta.tasso@istge.it (R.T.)

**Keywords:** stem cells, paracrine effect, secretome, microvesicles, trophic factors, tissue repair

## Abstract

For a long time, the stem cell regenerative paradigm has been based on the assumption that progenitor cells play a critical role in tissue repair by means of their plasticity and differentiation potential. However, recent works suggest that the mechanism underlying the benefits of stem cell transplantation might relate to a paracrine modulatory effect rather than the replacement of affected cells at the site of injury. Therefore, mounting evidence that stem cells may act as a reservoir of trophic signals released to modulate the surrounding tissue has led to a paradigm shift in regenerative medicine. Attention has been shifted from analysis of the stem cell genome to understanding the stem cell “secretome”, which is represented by the growth factors, cytokines and chemokines produced through paracrine secretion. Insights into paracrine-mediated repair support a new approach in regenerative medicine and the isolation and administration of specific stem cell-derived paracrine factors may represent an extremely promising strategy, introducing paracrine-based therapy as a novel and feasible clinical application. In this review, we will discuss the regenerative potential of fetal and adult stem cells, with particular attention to their secretome.

## 1. Introduction: From the Stem Cell Genome to the Stem Cell Secretome

Stem cell therapy has been shown to promote a substantial regenerative effect on damaged tissues and organs in several animal models. A large number of studies have provided strong evidence that stem cells (and in particular mesenchymal stem cells, MSC) can mediate tissue repair through the modulation of the local environment, influencing the immune/inflammatory response, sustaining angiogenesis and establishing co-operative effects with the resident cells, overall resulting in a significant cytoprotective and pro-survival beneficial influence [1,2]. The mechanism through which this regenerative result is achieved seems to rely more on the secretion of specific bioactive factors, rather than the direct differentiation of the transplanted stem cells in the host tissue, given the low incidence and poor efficiency of their survival and therapeutically relevant level of engraftment. This hypothesis is defined as paracrine effect and the composite set of the cytokines, chemokines and growth/trophic factors orchestrating many biological activities and secreted from stem cells (or shed from their membranes) is defined as secretome.

Direct evidence that stem cell-derived secretome plays a key role in mediating the regenerative effects observed *in vivo* has been demonstrated by a consistent body of studies on cardiovascular, renal, liver and lung injury, as well as in neurodegenerative disease models [3,4,5,6,7]. As proof of principle, some reports have demonstrated that the administration of stem cell-conditioned medium, which contains all the bioactive factors released by the cells in culture, can exert the same regenerative effect obtained with cell transplantation. Hence, current interest towards investigating the intercellular interactions underlying the paracrine effect is driving attention from the stem cell genome to the stem cell secretome, focusing on the cell-to-cell communication mechanisms.

In this scenario, microvesicles have been described as key regulators of the stem cell paracrine activity. The term of extracellular microvesicles (MVs) was first introduced to indicate nano-sized bodies released as shedding vesicles by various cell types into the extracellular environment. They include: (i) Exosomes, which are 30–100 nm diameter vesicles of endocytic origin obtained upon fusion of multivesicular bodies (MVB) with the cell membrane; (ii) Ectosomes (shedding vesicles), which are 100 nm–1 μm diameter vesicles directly shed from the cell membrane; and (iii) Apoptotic blebs, 1–5 μm diameter vesicles secreted by cells undergoing apoptosis [8]. Some confusion still exists in the literature regarding the distinction between exosomes and MVs. The difference between these two terms is based on the vesicle size: Exosomes are within 100 nm while microvesicles range from 100–1000 nm, but because this is still quite a novel research field, these definitions are flexible [9]. Microvesicles were first identified in sheep reticulocytes and described later on as mediators of the communication and activation processess involving B-lymphocytes and T-cell [10,11]. MVs were shown to be secreted by a variety of stem and somatic cells, either constitutively or when stimulated during activation or apoptosis; as well, they can be found in most of the physiological body fluids [10,11,12]. In recent years, exosomes have been specifically characterized with parameters other than their diameter size, such as the presence of a bi-lipid membrane similar to the plasma membrane, a specific flotation density of 1.1–1.18 g/mL on a sucrose gradient and an evolutionarily conserved set of markers including molecules from the tetraspanin family (such as CD81, CD63, CD9) and others like Alix, as well as cell type-specific antigens derived from the parental cell they originate from [13]. More recently, MVs, and in particular exosomes, have been described as playing a pivotal role in inter-cellular communication between stem cells and injured cells via paracrine signalling [12]. Exosomes were demostrated to contain proteins, bioactive factors, mRNAs and microRNAs reflecting the functionality of the cell producing them; they can transfer their content into recipient cells, resulting in the modulation of their protein synthesis and they were shown to act as carriers of the active component of the stem cell-conditioned medium and vehicles of the paracrine factors influencing the responder cells. As a matter of fact, MVs and exosomes, derived from stem cell-conditioned medium, exerted a beneficial influence, which is comparable to the regenerative effects obtained with stem cell transplantation in several preclinical disease models [14]. MVs and exosomes have recently captivated attention from the research community because of their paracrine factors content, thus suggesting them as a new therapeutic delivery tool. In this scenario, the fact that MSC, among many other stem cell types, are known to secrete MVs and exosomes provide a good rationale for testing their therapeutic potential in different animal models; thus, the analysis of the stem cell secretome and their MVs/exosomes’ content is a fast developing field, fuelled by a growing interest towards the clinical potential of this new, promising strategy for regenerative medicine.

In this review, we will discuss the latest and more relevant findings on the paracrine potential of the fetal and adult stem cells’ secretome for tissue regeneration and repair.

## 2. Paracrine Potential of Fetal Stem Cells

Stem cells are undifferentiated cells characterized by the capability of producing new cells with equal stem functionality (by self-renewal) or with restricted more specialized features via the expression of a specific committed phenotype (by differentiation) [15]. According to their origin and differentiation properties, they can be distinguished into embryonic stem cells, which are pluripotent, giving rise to all derivatives of the three primary germ layers and with unlimited self-renewal potential, or adult stem cells, which are found in several somatic tissues in the adult organism and are multipotent, with more restricted self-renewal potential [15,16]. 

The possibility of deriving multi- and pluripotent cells from fetal tissues, with properties intermediate between embryonic and adult stem cells, has recently been described by several groups [17]. Fetal tissue can be an attractive source of stem cells for therapy because of its pluripotency, proliferative ability and lack of immunogenicity. It could be obtained from a direct biopsy of the fetus during gestation or from fetal annexes such as umbilical cord blood, term placenta, villi and amniotic fluid; as the first procedure is associated with a defined morbidity/mortality and ethical issues, the other sources are preferred and represent an easily accessible and abundant font of progenitors. Stem cells with a therapeutic potential for regenerative medicine have been identified in term placenta, umbilical cord blood and amniotic membrane and fluid [18,19,20,21]. Particularly, human umbilical cord blood and placenta are well known sources of stem cells with potentials similar to bone marrow-derived mesenchymal stem cells [18,22] and they have been recently described as actively engaged in tissue repair mechanisms via the secretion of therapeutic bioactive factors. Human multipotent placenta mesenchymal cell-conditioned medium was demonstrated to contain paracrine factors from the IL6 superfamily and significant levels of pro-angiogenic bioactive molecules, such as vascular endothelial growth factor (VEGF), hepatocyte growth factor (HGF), basic-fibroblast growth factor (bFGF), transforming growth factor-β (TGF-β) and insulin-like growth factor-1 (IGF-1). The therapeutic relevance of their secretome has been investigated in several preclinical studies, showing a significant cytoprotective potential in a model of oxidative stress on placental endothelial cells, via activation of anti-apoptotic IL6ST/STAT3 and superoxide dismutase 2 (SOD2) expression in the responder cells; similarly, human MSC demonstrated to produce exosomes under hypoxic conditions, resulting in endothelial cell migration and tube formation while improving the wound healing and vascular regeneration process in a diabetic mouse model [23,24,25]. Human placenta and amniotic membrane-derived mesenchymal stem cells (AMSCs) have been shown to possess peculiar modulatory properties as well, such as the regulation of T-cell proliferation and dose-dependent inhibition of peripheral blood mononuclear cell-mediated immune responses [26,27]. In a preclinical transgenic model of Alzheimer’s disease, these cells also demonstrated long-term beneficial effects following transplantation, suggesting a significant paracrine role in re-instructing the host compromised immune system and secreting amyloid beta-degrading enzymes and high levels of TGF-β and matrix metallopeptidase 9 (MMP9) [28]. Therapeutic transplantation of cord-blood derived progenitors has been broadly described in different ischemic and oxidative stress models with encouraging results. Conditioned medium from human cord blood-derived endothelial stem cells were shown to exert mitogenic and chemotactic effects on keratinocytes and fibroblasts, possibly mediated by the significant level of the platelet-derived growth factors (PDGF)-α, PDGF-β and of the keratinocyte growth factor (KGF) cytokines strongly expressed in it. The paracrine factors released by umbilical cord blood-derived progenitor cells confirmed their beneficial potential also by accelerating the wound healing and the neovascularization process while exerting a renoprotective effect in a diabetic mouse model [29,30]. Another interesting source of human fetal MSC is represented by the perivascular connective tissue of the umbilical cord, the Wharton’s jelly [31]. These mesenchymal progenitors, defined as human umbilical cord perivascular cells (HUCPVCs), were demonstrated to exert significant proliferative effects on primary cultures of neurons and glial cells and a remarkable neuroprotector influence following transplantation into animal models of spinal cord injury and Parkinson’s disease. Their paracrine potential was mainly expressed via the increase of human neutrophil-activating protein-2 (NAP-2), neurotrophin-3 (NT-3), bFGF and glial derived neurotrophic factor (GDNF) at the site of injury [32,33]. Recent studies have further confirmed the peculiar potential of the HUCPVC secretome, showing that these stem cells preferentially express factors related to neuroprotection, neurogenesis and angiogenesis, with a dramatic influence on the metabolic viability of hippocampal neurons via the robust expression of nerve growth factor (NGF) [34,35]. HUCPVC have also shown therapeutic benefits when transplanted into a mouse preclinical model of splinted wound, as they accelerated the wound healing process by supporting collagen deposition and angiogenesis by paracrine mechanisms. Likewise, treatment with their conditioned medium alone provided similar results as stimulating anti-inflammatory M2 macrophages and sustaining angiogenesis through the expression of tissue-repairing cytokines like interleukin (IL)-10, transforming growth factor (TGF)-β1, vascular endothelial growth factor (VEGF)-1 and angiopoietin-1 at the healing wound site [36]. Furthermore, human stem cells derived form the umbilical cord perivascular fraction (PCs), together with mesenchymal stem cells from the cord blood, also demonstrated regenerative properties by mediating short- and long-term therapeutic benefits in a paracrine manner in a bronchopulmonary dysplasia model. The cells conditioned medium was shown to prevent the arrest of lung angiogenesis and to support the alveolar architecture in neonatal rats exposed to hyperoxia, thus preserving lung function at six months [37]. In particular, the remarkable cytoprotective effect of the exosomes isolated from human umbilical cord-MSC has also been recently described, showing that these microvesicles can exert an important therapeutic benefit in ameliorating oxidative stress, improving cell survival and reducing apoptosis and necrosis in the proximal kidney tubules of a rat model of cisplatin-induced nephrotoxicity [38].

Along with fetal tissue, amniotic fluid (AF) is another source of stem cells with suitable potential for therapeutic applications [39]. Amniotic fluid is known to contain multiple cell types derived from the developing fetus and it represents an alternative source of immature progenitor cells that can be easily collected during amniocentesis, a well established technique for prenatal diagnosis with low risk both for the foetus and the mother [40], as well as during C-section procedures at term. In the last few years, several groups have presented various studies demonstrating the presence of heterogeneous progenitors in the amniotic fluid, mainly with mesenchymal characteristics [41,42,43,44,45]. In particular, pluripotent stem cells from the amniotic fluid (AFS, Amniotic Fluid Stem cells) can be isolated using discarded back-up amniocentesis samples by selection for the expression of the membrane stem cell factor receptor c-kit [46]. AFS share some embryonic stem cell properties, such as the expression of pluripotency stem markers like SSEA4 and OCT4, high proliferative and self-renewal potential and *in vitro* differentiation into adipogenic, osteogenic, myogenic, endothelial, neural and hepatic lineages; furthermore, they were recently demonstrated to give rise to induced pluripotent stem cells (iPS) via non-viral methods [46,47]. Recently, a growing body of work has suggested a therapeutic role for the secretory profile of these cells. The first detailed description of the paracrine potential of the amniotic fluid mesenchymal stem cells comes from a study on skin wound repair, describing the cells-conditioned medium as a powerful source of a broad array of cytokines and chemokines, such as IL-8, IL-6, TGF-β, tumor necrosis factor receptor I (TNFRI), VEGF and EGF, with relevant roles in instructing dermal fibroblasts during wound healing, following activation of the TGF-β/SMAD2 pathway [48]. The human c-kit+ AFS cell secretome has been also partially characterized, showing a significant enrichment for pro-angiogenic soluble factors, such as monocyte chemoattractant protein-1 (MCP-1), stromal cell-derived factor-1 (SDF-1) and VEGF in a dose-dependent manner. Indeed, the secretome of these cells provided a remarkable paracrine effect *in vivo* in a limb ischemia-reperfusion rmouse model, by enhancing vasculogenesis via chemo-attraction of host endothelial precursor cells [49]. These results were further confirmed in a another preclinical ischemic model of fascio-cutaneous flap where the topical administration of human c-kit+ AFS cell-conditioned medium provided significant improvement in the perfusion level of the injured area, together with recruitment of local progenitor cells and subsequent differentiation into endothelial lineage with capillary formation [50]. Moreover, the amniotic fluid stem cell secretome proved to promote tissue repair not only by mediating pro-angiogenic regenerative effects, but also via the local and systemic modulation of inflammation, as shown in a mouse model of acute hepatic failure. Indeed, in this work, the AF mesenchymal stem cell secretory profile showed to be significantly enriched for a variety of interleukins (IL-10, IL-27, IL-17E, IL-12p70, IL-1β and IL-1ra) and liver regenerative mediators (MCP-1, SDF-1, platelet-derived endothelial cell growth factor (PD-ECGF), tissue inhibitors of metalloproteinase, TIMP1 and TIMP2, fibroblast growth factor 7 (FGF7), and EGF) [51]. The cytoprotective paracrine influence of the AFS cells was also validated in other injury preclinical models of lung, renal, intestine and heart diseases [52,53,54,55,56]. Of particular interest, human AFS cells and their conditioned medium were shown to exert a remarkable pro-survival and anti-apoptotic effect on the ischemic cardiac tissue in an experimental model of acute myocardial infarction in rat, resulting in a significant decrease of the infarct size and cardiomyocyte death within two hours of treatment with a putative paracrine effector identified in thymosin beta 4, a cardioprotective factor actively secreted by these cells [55]. Similarly, remarkable results were obtained in a study evaluating the regenerative role of rat AFS cells in rat neonates affected by necrotising enterocolitis. Cell transplantation and the injection of their conditioned medium resulted in modulation of the cyclooxygenase-2 enzymatic activity in the host stromal cells, causing a decrease of bowel inflammation, tissue necrosis and damage with significant improvement of intestinal function [56]. A well-characterized regenerative role for the amniotic stem cell-derived exosomes has also been reported using an *in vitro* co-culture system with human amniotic mesenchymal stem cells and primary fibroblasts from a patient affected by cystinosis with a lysosomal cystine transporter mutation (CTNS −/−). In this study, the amount of altered cell cystine detected in culture was significantly reduced, suggesting a paracrine effect of the amniotic stem cells on the mutant fibroblasts, which was mediated by the microvesicles derived from the stem cells and transferring wild type cystinosin and CTNS transcript to the affected target cells [57]. 

In light of these considerations, fetal stem cells possess a promising paracrine capacity which may be the principal mechanism contributing to tissue repair, in addition to their multipotent differentiation potential. Considering their remarkable *in vitro* self-renewal properties, these stem cells may represent an ideal candidate for paracrine therapy, in order to isolate and scale-up putative drug-like formulation from their secretome for future regenerative medicine applications. 

## 3. Paracrine Potential of Adult Mesenchymal Stem Cells

Adult stem cells are involved in the maintenance of homeostasis and physiological repair of somatic tissues and organs through a fine balance between self-renewal and differentiation; they reside in specialized niches, a term used to describe the complex interaction of different cells, matrix components and biological factors in distinct tissue locations [58]. After an insult, stem cells can actively exit the niche and become activated in order to mediate tissue regeneration; in particular, activated stem cells have been demonstrated to secrete immunomodulatory and trophic factors, which can support the repair process by endogenous mechanisms [59]. Among the different adult stem cell populations described so far, bone marrow-derived mesenchymal stem cells (BM-MSC) have been broadly described as a clinically effective therapeutic agent for a variety of tissue injuries. MSC are routinely derived from primary cultures of adult tissues, such as bone marrow and adipose tissue, along with other stromal and mesodermal sources [60]. According to the International Society for Cellular Therapy (ISCT), adult MSC can be characterized based on their specific culture conditions and immunophenotypic features, along with their mesodermal differentiation potential. These criteria include the expression of the mesenchymal markers CD105, CD73 and CD90 with lack of mature hematopoietic antigens such as CD45, CD34, CD14, CD11b, CD79a, CD19 and HLA-DR and the *in vitro* potential to give rise to adipogenic, chondrogenic and osteogenic lineages [61]. Nevertheless, a clear cell surface marker to identify a true MSC has not been identified yet. In addition to their multipotent transdifferentiation potential, adult MSC have also been shown to play a key role as cellular modulators in tissue repair mechanisms. An increasing body of work based on lineage trace studies has showed that transplantation of adult MSC can exert a remarkably beneficial effect in different injury models, despite their poor engraftment and *in vivo* low level of differentiation in the long term. Such a therapeutic effect seems to be mediated by the MSC paracrine potential to secrete trophic factors, which sustain local neovascularization, inhibit cell death with modulation of the immune response and mobilization and survival of host cells [62,63]. In a rat model of myocardial infarction, transplantation of allogeneic MSC demonstrated beneficial effects through their paracrine activity, improving left ventricular function at four weeks after transplantation [64]. The cardioprotective role of the adult MSC secretome was further confirmed by exposing cardiomyocytes to genetically modified MSC-conditioned medium under hypoxic conditions [65]; in particular, MSC-conditioned medium also promoted *in vitro* proliferation and migration of endothelial cells in a dose-dependent manner with VEGF and bFGF being the key factors implicated in this process. Likewise, in a murine model of hindlimb ischemia, the factors released into the adult MSC-conditioned medium enhanced collateral flow recovery and remodeling, thus attenuating muscle atrophy [2]. An additional confirmation of the therapeutic role of the MSC secretome has also been reported in a preclinical model of acute renal failure, where cell transplantation improved the general outcome via paracrine effects influencing the modulation of the inflammatory, vascular and apoptotic/necrotic processes related to the ischemic kidney injury. Notably, none of the transplanted MSC differentiated into mature tubular or endothelial phenotypes, while the expression of pro-inflammatory molecules such as IL-1β, TNF-α, IFN-γ and of inducible nitric oxide synthase were significantly reduced in the treated kidneys, with concurrent induction of the anti-inflammatory cytokines IL-10, bFGF, TGF-α, and Bcl-2 [66]. In light of these results, the characterization and therapeutic use of MSC, with respect to their ability to produce and secrete bioactive factors, inspired their description as “site-regulated, multidrug dispensaries, or injury drugstores” [67]. 

It has also been recently demonstrated that the intrinsic capacity of MSC to activate endogenous regenerative mechanisms and to induce the mobilization of host cells is critically dependent on their commitment level, highlighting the importance of carefully investigating the differences in the soluble morphogens used in the culture conditions. The addition of basic-fibroblast growth factor (bFGF or FGF-2) to primary bone marrow-derived MSC cultures proved to be a key element in order to select *in vitro* specific MSC subpopulations with potential to induce the host regenerative response *in vivo*. Implantation of constructs seeded with bFGF-selected MSC led to formation of bone of host origin through the activation of an endochondral ossification process, whereas an intramembranous ossification directly performed by the seeded cells was observed in implanted constructs with unselected MSC [68]. Moreover, bFGF-selected mouse MSC, when ectopically implanted in immunocompetent syngeneic hosts were demonstrated to exert a potent anti-inflammatory effect towards mobilized macrophages through the release of prostaglandin E2 (PGE2); MSC-released PGE2 induced a functional switch of macrophages from a pro-inflammatory to a pro-resolving phenotype and this event triggered a cascade of cellular events resulting in the creation of a bone regenerative niche via the mobilization of host bone marrow-derived endothelial and osteogenic cells [69]. A similar anti-inflammatory effect was also described in a mouse model of sepsis, where systematically injected MSC induced IL-10 production in host macrophages [70]. Moreover, it has been reported that intravenous (iv) or intraperitoneal (ip) injection of MSC resulted in a substantial paracrine effect, reducing inflammation and increasing vascularization in a rat model of sterile injury to the cornea: Transplanted MSC reduced the damage to the cornea, without showing significant engraftment in the host tissue, suggesting that their pro-resolving effect was mainly due to the secretion of TNF-α stimulated gene/protein 6 (TSG6) [71]. 

It is well known that the extracellular microenvironment contains solutions of proteins, polysaccharides as well as vesicles containing proteins, mRNA, and micro-RNA [72]. Recent reports identified in the release of MVs and exosomes a novel and co-operative paracrine mechanism carried out by adult MSC [12,73]. The first study that investigated the therapeutic effect of adult MSC-derived MVs was performed in a rat model of a glycerol-induced acute kidney injury. In this work, systemic injection of MVs induced epigenetic changes in the resident host cells by horizontal transfer of mRNA, leading to cell cycle restoration and activation of tissue regenerative programs [74]. In a follow-up study, the same group also demonstrated that, immediately after ischemia and reperfusion injury, a single injection of MVs obtained from adult MSC could prevent both acute and chronic kidney disease in a rat model [75]. Moreover, recent studies proposed that the beneficial paracrine effect observed on cardiovascular cells in several preclinical models of cardiac ischemia and/or disease following MSC transplantation, might be mainly mediated by these stem cell-derived MVs. In a preclinical model of myocardial ischemia/reperfusion (I/R) injury, the injection of human MSC-conditioned medium led to a 60% reduction in the myocardial infarct size, showing that only the fraction of the conditioned medium containing products in the range of 100–220 nm size provided cardioprotection [76]. A more recent study also highlighted the cardiac regenerative properties of human MSC-exosomes, showing that, in a mouse model of cardiac I/R injury, the treatment with intact exosomes resulted in the restoration of bioenergetics, reduction of oxidative stress and activation of pro-survival signaling pathways, overall enhancing the cardiac function [77]. The therapeutic effect of MSC-derived MVs was further confirmed in other animal models of acute myocardial injury, where their administration resulted in a cardio-protective effect dependent on the release of cytokines, bioactive molecules and growth factors, mediating regenerative effects similarly to MSC or MSC-conditioned medium transplantation [12]. 

MVs and exosomes released by MSC are currently being tested in several other preclinical injury models, such as stroke [78], liver fibrosis [79], rheumatic diseases [80], and acute lung injury [81]. The wide range of therapeutic effects mediated by MSC-derived trophic factors and MVs/exosomes suggests that their potential for clinical applications is much broader than those currently identified. Hopefully, scientific advances in the understanding of the paracrine properties of MSC will pave the way to innovative clinical strategies, where stem cell-based paracrine therapy could actively contribute to regenerate/repair the appropriate tissue, with adult MSC already a broadly investigated candidate for clinical trials and regenerative medicine strategies.

## 4. Tissue Regeneration by Paracrine Effect via Re-Activation of Resident Progenitors

Various adult and fetal progenitor cells have been demonstrated to exert cytoprotective, pro-survival, anti-inflammatory, anti-apoptotic and angiogenetic effects on injured cells by secreting and delivering regenerative factors within MVs/exosomes. More recently, another mechanism underlying the paracrine hypothesis has been proposed, suggesting that the bioactive factors secreted by the exogenous transplanted stem cells, and contained in their conditioned medium, might act not only on the injured host cells, but also by targeting an endogenous progenitor population residing in the tissue, in order to recruit and activate them to initiate/sustain the repair process, overall improving the functional outcome by autocrine effects. This synergistic result, which is based on the intercellular crosstalk via paracrine signalling between the exogenous stem cells and the host endogenous progenitors, has been described in different cell therapy preclinical studies, in particular in cardiovascular, neural, bone and revascularization models. The restoration of cardiac progenitor cell (CPC) populations and the activation of the molecular pathways within their repair programme have been recently described following the paracrine effect mediated by transplanted adult bone marrow-derived stem cells in the setting of myocardial ischemic injury [82,83,84,85]. This approach resulted in two mechanisms of endogenous cell proliferation, targeting both CPC and the surviving pre-existing cardiomyocytes [86]. Transplantation of bone marrow MSC was also shown to promote tissue repair through secretion of paracrine factors and reactivation of local progenitors in an experimental model of glaucoma, as well as in an ectopic bone regenerative study [69,87,88]. Likewise, fetal stem cells such as AFS cells have been shown to trigger a chemo-attractive response resulting in the recruitment of CD31+/VEGFR2+ and CD31+/CD34+ host endothelial progenitor cells into ischemic subcutaneous tissues in rats and of mesodermal precursors committed to fat, muscle, fibrous tissue and immature bone lineages, following subcutaneous implantation onto hydroxyapatite scaffolds into nude mice [50,89]. 

Hence, these observations support the developing idea that harnessing the stem cell secretome can lead to a double beneficial influence on the injured tissue (1) by direct modulation of the local environment, thus providing an immediate cytoprotective, angiogenic and anti-inflammatory effect during the acute phase following injury and (2) by boosting the local resident stem/progenitor cells, with the aim of achieving a more pronounced tissue-specific repair programme and instructing the stimulation of resident endogenous progenitors as a critical mechanism for future development of pharmacological paracrine therapy.

## 5. Tissue Regeneration by Paracrine Action: Microvesicles as Shuttles for Small RNAs

An additional feature of the paracrine therapy is represented by the characterization of the signalling mechanisms that orchestrate the activity of the stem cell secretome, and in this scenario, MV/exosome secretion represents a crucial aspect. Microvesicles and exosomes currently represent a very intriguing and timely focus in stem cell biology, as they are considered the key component mediating stem cell-derived paracrine effects on target cells via cell-to-cell signalling. Different studies have described them as active components of the pro-angiogenic and regenerative effects exerted by stem cells in animal models of myocardial ischemia/reperfusion injury or corneal assay [12,90,91,92,93,94]. Their role as paracrine effectors is based on evidence that they can transfer—locally or through biologic fluids—not only bioactive molecules, but also genetic information between the secreting and the responding cell; therefore, they are emerging as interesting mediators of cell-to-cell communication, either via proteomic or genomic interactions [95]. Several independent studies have recently reported that MVs and exosomes can be involved in the transfer of genetic material in the form of mRNAs and microRNAs (miRNAs). miRNAs are a novel class of small non-coding post-transcription regulating RNAs, which can be involved in cell proliferation, differentiation, development, and death. A growing body of work supports the idea that stem cell-derived MVs/exosomes can modulate or reprogram the phenotype of a recipient somatic cell via the horizontal transfer of small RNAs, in particular of miRNAs [95,96,97]. Therefore, it might be reasonable to assume that stem cells can exert their regenerative effects on the injured tissue by paracrine mechanisms via delivering certain transcripts which can up- or down-regulate specific pathways in the target cells, such as local cell de-differentiation and cell cycle re-entry, while promoting cell survival, angiogenesis and endogenous tissue repair activation. This hypothesis has been validated in several preclinical studies. In an experimental animal model of renal ischemia/reperfusion injury, adult endothelial progenitor cell-derived MVs were shown to restore the angiogenic program in quiescent cells by the horizontal transfer of mRNA and to prevent acute kidney injury via a mechanism involving the angiogenic miRNAs miR-126 and miR-296; the renoprotective effect achieved with the microvesicle treatment was lost after treatment with RNAse or transfection with specific miR-antagomirs, suggesting a key role of the miRNAs shuttled by the MVs in this regenerative process [98,99]. Similar results were obtained in a murine model of hindlimb ischemia, where treatment with the MVs isolated from adult stem cells and containg miR-126 and miR-296 resulted in significantly enhanced perfusion and reduced damage of the muscle [100]. More recently, adult MSC were demonstrated to be able to communicate with brain parenchymal cells and to influence neurite outgrowth by transfer of miR-133b via exosomes; loss of expression of miR-133b in the MSC and luciferase assay confirmed that the results obtained in the study were attributed to the delivery of functional miRNAs from the MSC-derived exosomes to the neural cells [101]. Adult rat MSC overexpressing GATA-4 also showed to mediate cytoprotective and pro-survival effects on hypoxic cardiomyocytes via a miRNA-associated mechanism, in which the expression of the anti-apoptotic miR-221 transferred by MVs significantly enhanced cardioprotection by reducing the levels of p53 in the treated cells [102]. By acting as a shuttle for the transfer of genetic information, stem cell-derived MVs/exosomes play a critical role in the intercellular communication phase of the paracrine effect. Since miRNAs are post-trascriptional regulators inducing epigenetic changes in the target cells that survive the injury, they can be envisioned as a novel strategy to modulate and activate the tissue endogenous regenerative programme.

## 6. Moving Forward into the Clinical Scenario: From Stem Cell Therapy to Paracrine Pharmacological Therapy

In the last few years, significant effort has been invested in stem cell-based therapy for tissue regeneration following injury, trauma and chronic disease. However, much scepticism still surrounds cell transplantation- and tissue engineering-based methods, due to their poor clinical feasibility, namely limitations associated with safety, immunogenicity, and, in many cases, limited efficacy of the injected cells. A strong limiting factor to stem cell-base therapy is also represented by the high cost of the procedure to treat each patient that precludes application to a large number of people within public and private health social systems. 

In this scenario, isolation and administration of specific stem cell-derived regenerative factors for future paracrine pharmacological therapy may represent an extremely promising strategy with the clinical advantage of limiting many of the safety concerns associated with the transplantation of viable replicating cells. Indeed, this approach represents a more tractable model to still obtain stem cell-mediated regenerative effects but through the cell-free delivery of soluble molecules, shifting attention from cell therapy to paracrine pharmacological treatment. Insights into paracrine-mediated repair support a new approach for regenerative medicine, via the identification of suitable cocktails of stem cell-derived trophic factors to trigger endogenous tissue repair mechanisms, thus introducing paracrine therapy as a novel and feasible clinical read-out for patients suffering from injuries and traumas. In addition, patients suffering from diseases such as ischemic injuries and/or traumas need prompt therapeutic intervention. Therefore, it would be ideal to have access to “off-the-shelf” products ready for administration on demand, and by taking advantage of our knowledge of the stem cell secretome, we could envisage the use of stem cells as “drug-stores” to produce specific soluble paracrine factors, which can ultimately be administered orally or systemically. As well, this strategy removes the invasive or additional surgical procedures generally required for cell-therapy or tissue engineering methods, together with the risks associated with them. 

While paracrine therapy may represent a novel “cell-free” approach for tissue regeneration, it is worth considering that cytokine therapy strategies, based on the delivery of multiple/high doses of a single trophic factor, have not yet met expectations, due to problems related to appropriate delivery, pharmacokinetics and *in vivo* stability of such molecules. Therefore, it is crucial to optimize the stem cell culture protocols *in vitro*, in order to produce the right amount, concentration and combination of paracrine factors and to obtain the required therapeutic effect once administrated *in vivo* [103].

In this scenario, MVs and exosomes may represent an ideal vehicle for paracrine therapy, since they can be used as immunologically inert nano-carriers with the intrinsic capacity to carry a significant amount of stem cell-derived bioactive molecules (proteins, lipids, mRNAs and miRNAs), while protecting these from the degradative enzymes/chemicals and bypassing biological barriers for the *in vivo* targeting of specific tissues. As a matter of fact, exosomes show several features of the ideal drug carrier, such as membrane penetration potential, intrinsic homing ability, long circulating half-life and the possibility of undergoing membrane modifications in order to enhance targeting of specific cell types [92]. Despite their promising and appealing therapeutic potential, the biological characterization of the stem cell-derived MVs and exosomes still represents a novel approach in regenerative medicine and the necessary proteomic and genomic data are either still lacking or requiring more comprehensive analysis. Currently, only one detailed proteomic analysis of human adult MSC-derived MVs has been reported, identifying about 730 MV proteins, including 43 surface receptors and signaling molecules that control self-renewal and differentiation of MSCs. The results of this analysis suggested that MV protein content might relate to cellular processes such as cell proliferation, adhesion, migration, and morphogenesis [104]. mRNA and miRNA microarray characterisation of MSC-derived MVs is another exploratory approach which is fastly developing [9]. One of the first studies using human embryonic stem cell-derived MSC and based on the detailed microarray analysis of their MVs described a shared expression in the MV miRNA content with that of the parent cells [97]. In a similar study, the RNA contained within the MVs secreted by human bone marrow-MSC and liver resident stem cells was profiled for 365 known human miRNAs. The analysis resulted in 41 miRNAs co-expressed in the MVs and in the parental cells, which could be involved in multi-organ development, cell survival, differentiation and, to some extent, immune system modulation [105]. Due to the enormous potential of paracrine factors and MVs released by MSC for future regenerative therapeutic purposes, there is an urgent need for a detailed identification of such elements, and the exponential increase in exosomal studies witnessed in recent years has required the scientific community to have access to reliable protocols for exosomal isolation and purification procedures. In order to provide such information, ExoCarta, a manually curated web-based database, has been recently created as a catalogue of exosomal proteins, RNA and lipids [8]. With this bioinformatic tool, the information on exosomal isolation/purification procedures, samples used and exosomal molecular components, such as proteins, mRNAs and miRNAs reported in previously published studies, can be easily obtained. Similarly to ExoCarta, another online dataset of extracellular vesicles, defined as Vesiclepedia, will be soon available for consultation, and with the assistance of the International Society of Extracellular Vesicles (ISEV), the current confusion on the definition of MVs and exosomes should be resolved by the introduction of a standardised nomenclature, in association with stringent purification protocols for exosome isolation [106].

**Figure 1:**
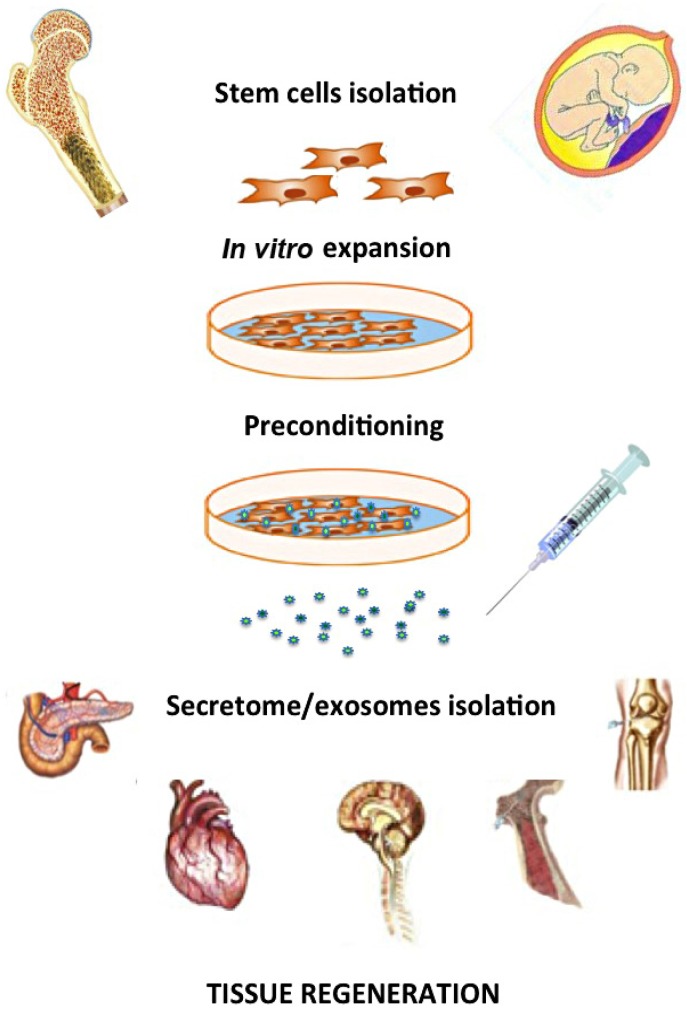
Schematic representation of paracrine therapy using adult or fetal mesenchymal stem cells. After isolation, stem cells are expanded *in vitro* and stimulated in order to release trophic factors into their conditioned medium via microvesicles/exosomes secretion. Factors directly released in the conditioned medium or contained in the microvesicles may be further isolated and use as therapeutic agent for tissue regeneration and repair.

Another crucial aspect to be evaluated, when harnessing the stem cell secretome for therapeutic purposes, is defining suitable strategies to stimulate the cells to release significant amounts of the desired paracrine factors in their conditioned medium or microvesicles, in order to provide tissue regeneration, as represented in Figure 1. Most of these approaches are performed *in vitro* and are referred to as preconditioning methods [103]. Serum deprivation and starvation have been broadly described in the recent literature as common strategies to induce the secretion of paracrine factors and MVs from cultured stem cells. For example, bone marrow MSC cultured under these stress conditions were shown to undergo epigenetic changes and to significantly upregulate the expression of the pro-angiogenic and anti-apoptotic factors IGF1 and leptin [107]. Moreover, the medium conditioned by MSC cultured under serum-deprived conditions denoted an angiogenic potential higher than that obtained in standard conditions in an *ex vivo* rat aortic and in a chick chorioallantoic membrane angiogenesis assay [108]. To mimic the *in vivo* ischemic environment, in which transplanted stem cells are likely to exert their beneficial effect, hypoxia preconditioning has been broadly used by subjecting MSC to physiological conditions of low oxygen tension (<5%) *in vitro*. This strategy induced adult and fetal MSC to secrete higher levels of pro-angiogenic and pro-survival cytokines, such as VEGF and bFGF, to counteract hypoxic effects, while activating survival pathways with genes like Akt, HO-1 and Hsp70 [25,109,110]. Although preconditioning via hypoxia exposure was shown to significantly increase stem cell secretion of bioactive trophic factors, a general consensus on this method has not yet been obtained, as there is still considerable variation in the hypoxic protocols and results published so far. Further analyses are required to determine whether the hypoxia preconditioning results can be mantained in the long term in order to produce a clinically relevant result [103]. To improve the efficiency of the MSC secretome for cardiac repair, genetic manipulation has also been suggested, using transgenes for conditional overexpression of genes like Akt, IGF-1, VEGF GATA-4 and SDF-1. This approach was validated in an ischemic injury model, resulting in sustained paracrine effect mediated by trophic and angiogenic molecules secreted from MSC, along with mobilization of endogenous c-kit+ and CD31+ progenitors and reduced ventricular remodeling [111,112,113,114,115]. Despite the encouraging results achieved with this startegy, the sustained paracrine effects might be limited to the overexpression of a single specific gene, the expression levels of which might not correlate with the concentration of the desired secreted factors, in addition to the risk of creating secondary, undesired effects; moreover, the potential use of viral vectors can limit the clinical translation of this approach, given the safety concerns associated with this technique. Molecular stimulation of stem cells using a set of cytokines, chemokines and growth factors playing a key role during the inflammatory response or involved in the development of ischemic injury and disease, have also been used to trigger and sustain MSC paracrine secretion *in vitro*. Preconditioning MSC with pro-inflammatory cytokines such as TGF-α, TNF-α and lipopolysaccharide (LPS) resulted in an increased production of VEGF, IL-6, IL-8 and MCP-1 [116,117,118,119], while stimulation of mouse MSC with addition of bFGF in the culture medium resulted in selection of specific subpopulations with increased paracrine potential in terms of inducing the host regenerative process [68]. Similarly, co-colture with apoptotic cells has been adopted to prime MSC against apoptotic cytokines, in order to stimulate the secretion of pro-survival factors, such as STC-1 [120]. Nevertheless, more comprehensive studies are still needed to fully elucidate this approach, as the synergistic effects created *in vitro* using different cocktails of signalling cues in order to mimick the injury setting might differ from the actual situation *in vivo*. A further relevant point to be considered for future stem cell-derived paracrine therapy regards the use of primary stem cells to isolate specific therapeutic factors. As a matter of fact, adult and fetal MSC (although the latter to a lesser extent) are characterised by significant, yet limited, proliferative and self-renewal potential. Thus, a scale-up production of MSC-derived paracrine factors for future applications would involve the use of repeated isolation of cells with the risk of batch-to-batch variation [92]. In order to avoid the finite expansion capacity of MSC and to improve the yield of the secreted regenerative factors, immortalization of primary human stem cells has been recently suggested. Human embryonic stem cell-derived MSCs were transfected with a *MYC* gene to demonstrate that, once immortalized, they can increase their proliferative rate, while maintaining the production of exosomes therapeutically effective in reducing the infarct size in a mouse model of cardiac I/R injury [121].

Lastly, the technology available to analyse the stem cell secretome will be an instrumental role for future paracrine therapy, since the cell culture medium conditioned by adult and fetal MSC is a subject of intensive characterization in the search for specific released factors and MVs/exosomes that might be used for regenerative medicine purposes. In this scenario, the techniques applicable to stem cell secretomics mainly focus both on proteomic analysis and microarrays for small RNAs screening, coupled with bioinformatic computational approaches [122]. Proteomic investigation of MSC secretome is currently based on the analysis of cell-conditioned medium via protein/peptide separation techniques (such as 2-dimensional gel electrophoresis and liquid chromatography), followed by specific protein identification by mass spectrometry and immunological assays like ELISA (Enzyme-Linked ImmunoSorbent Assay) and western blotting. Recent advances in mass spectrometry have led to more specific techniques for relative protein quantification, such as, for example, the SILAC (Stable Isotope Labelling by Amino acids in Cell culture) methodology, which is based on the incorporation of the essential aminoacid arginine and/or lysine, which are labelled with the non-radioactive carbon-13, nitrogen-15 or deuterium isotopes into the newly synthesized proteins during cell culture [123]. This method can provide detailed comparison and quantification of protein levels between samples, thus offering more comprehensive data on the cell secretome profile. As well, antibody-based assays have been significantly improved to detect specific antigens and proteins that are present in the cell-conditioned medium at very low concentrations. Multiplex immunological assay, in the form of microarray or microbeads formulations like the Luminex^®^ methodology, have been recently developed in order to simultaneously measure several hundreds of compounds in multiple samples, with the possibility of detecting cytokine concentration less than 1 picogram/mL [122]. 

As for the proteomic characterization of the stem cell-derived MVs and exosomes, their profiling is based on the same techniques as for the measurement of soluble secreted peptides, such as one dimensional sodium dodecyl sulphate—polyacrylamide gel electrophoresis (1D SDS PAGE), liquid chromatography and mass spectrometry; at present, only a few studies have been published so far, such as those involving neural stem cells, adipose tissue and human embyonic stem cell-derived MSC [118,124,125]. MSC-derived exosomes have been shown to shuttle not only proteins but also small RNA between cells, resulting in the epigenetic modulation of the recipient cells [96]. The profiling of RNA content within the stem cell-derived exosomes has been mainly described through the use of molecular techniques, like microarray and qRT-PCR analysis [97,126]. Another critical tool for the proteomic and genomic profiling of the stem cell secretome is represented by bioinformatics, which has undergone significant development in recent years. Several web databases and search engines have been recently created in order to interpret the large amount of data generated by the analysis of the MSC secretome. Software such as DAVID (the Database for Annotation, Visualisation and Integrated Discovery), the Ingenuity Pathway Analysis (Ingenuity^®^ Systems), Bioinformatic Harvester and the Panther (Protein Analysis Through Evolutionary Relationships) have been recently developed to provide clusterings of groups, maps of signalling pathways and entries for possible interacting proteins in order to predict the putative roles of the stem cell secreted factors in metabolism, inflammation, immune response, development, tissue repair and regeneration [122]. Although much progress has been made in terms of the technology and the tools currently available for the screening of the MSC secretome, a comprehensive database of their secreted paracrine factors and small RNAs has to be yet generated as reference. Moreover, it has to be considered that each technique presents some limits, either in the instrument sensitivity or in the specificity of the method itself; therefore, a more complete characterization of the MSC secretory profile will need a comprehensive combination of several different approaches at proteomic, genomic and computational levels.

## 7. Conclusions

Over the past few years, we have witnessed scientists and physicians expressing increasing enthusiasm for the therapeutic potential of stem cells. Traditional cell therapy is based on the belief that, when healthy cells are injected into patients, they can stimulate the body’s own healing process. Recent data available on database about clinical studies of human participants conducted around the world (Clinical Trials.gov, August 2013) showed that more of 4600 stem cell-based therapeutic approaches are globally active, and 300 of those are MSC-related. Based on advanced cell technology, promising curative effects and ethical issues, MSC have become the most common cell source in cell-based treatment. In light of these considerations, many biotechnology companies are supporting stem cell therapy and focusing on producing and commercializing human cell technology to treat degeneration of tissue and organs. 

Although much effort has been invested into preclinical research, several concerns and risks associated with the direct use of stem cells still need to be addressed. As well, many questions remain unanswered, such as the potential of the transplanted stem cells to generate fully functional new cells in the patient’s damaged organ. In addition, we also need to consider a manufacturing risk: Stem cell therapy requires Good Manufacturing Practice (GMP facility) with a consequent drastic increase in the costs of cellular technology. Thus, despite the extensive research developed in the last 20 years and the encouraging results obtained with cell therapy for the regeneration of tissue such as the epithelium (*i.e.*, skin repair following burns or cornea regeneration), many concerns still exist related to the clinical application of stem cells for the repair of mesodermal tissue and organs with many researchers currently suggesting that the use of MSCs should be reconsidered. The clinical potential offered by MSC for therapeutic applications includes diverse clinical targets, and in most of the current ongoing clinical trials, stem cells seem to mostly contribute in modelling the *in vivo* regenerative microenvironment by secreting bioactive molecules acting on angiogenesis, inflammatory and local immune response. 

Furthermore, to establish the basis of a future paracrine pharmacological therapy, it is of crucial relevance to identify the most suitable stem cell font. The ideal stem cell source should be selected upon consideration of their paracrine potential and the feasibility of their isolation, together with their *in vitro* self-renewal properties. Both adult mesenchymal stem cells and fetal stem cells unambiguously fulfil these criteria, as they can be easily isolated, expanded and cryopreserved while retaining a stable karyotype and low immunogenic profile. While adult stem cells (such as bone marrow mesenchymal stem cells or adipose stem cells) are isolated from biopsy obtained by surgical procedures, fetal stem cells (like amniotic fluid or umbilical cord stem cells) can be harvested from leftover samples from prenatal diagnosis or discarded tissue collected at birth, avoiding all the surgical risks associated with the first source, which might retain some level of morbidity. At the same time, despite the fact that fetal stem cells retain a more immature potential and higher self-renewal properties compared to progenitor cells from adult sources, the characterisation of their secretome is incomplete and further studies need to be performed.

Finally, although stem cell-derived paracrine therapy may represent an extremely exciting and novel therapeutic strategy, several aspects are yet to be addressed before clinical use of their secreted factors and MVs is considered. The large-scale production of specific paracrine molecules (proteins, lipids, small RNAs) obtained from cultured stem cells needs to be defined in detail; the regenerative potential of different preparations of stem cell-derived MVs and trophic factors has to be investigated and experiments to assess the long-term safety, bio-distribution and persistency of the therapeutic effects *in vivo* must be performed and evaluated accurately [127]. Furthermore, our knowledge of the MSC secretory profile is mainly based on *in vitro* studies, thus it has to be considered that the secretome of cells cultured under specific controlled conditions might be quite different in terms of composition, concentration and timing from that *in vivo*, where stem cells can modulate their paracrine potential in response to the stimuli released by the injured microenvironment. Hence, it will be particularly important to profile the MSC secretome *in vivo* and to develop more sensitive techniques in order to characterise the dynamic expression of MSC-secreted factors and their qualitative and quantitative changes in response to the local milieu.

In this review, we discussed how the number of studies exploiting fetal and adult MSC therapeutic potential based on the release of regenerative trophic factors, rather than their direct trans-differentiation capacity, is rapidly evolving including several clinical applications, such as graft versus host disease (GVHD), myocardial infarct, stroke, acute kidney, lung failure, wound healing, rheumatoid arthritis, and multiple sclerosis [67]. 

It is reasonable to conclude that the protective and restorative benefits mediated by adult and fetal stem cells are partially due to paracrine effects obtained by the release of specific cytokine, chemokine andgrowth factors, which are likely exerted via exosome and microvesicles secretion. At the same time, the advantage of using cell-based therapy relies on the possibility of providing the physiological concentrations of trophic factors to injured tissue at the appropriate timing; these synergistic effects may provide a regenerative microenvironment in the damaged tissue as well as recruit resident progenitor cells for tissue repair.

The paracrine potential of adult and fetal stem cells might be then considered as a new regenerative medicine approach for future clinical use, in order to cure specific disease or congenital defects.
